# Supplementary material for: Patterns of Intron Gain and Loss in Fungi
Source: PLoS Biol. 2004 Nov 30;2(12):e422. doi: 10.1371/journal.pbio.0020422 (PMC532390; doi:10.1371/journal.pbio.0020422)
Supplement: Table S1 — Also available at http://genes.mit.edu/NielsenEtAl/. (4.3 MB ZIP). [file pbio.0020422.st001.zip › NielsenEtAl/html/550.html]

AN1700.1.NCU09450.1.MG05991.1.FG07499.1


```
 CLUSTAL W (1.82) Multiple Sequence Alignments - Introns Inserted


Sequence 1: NCU09450.1	1188 aa
Sequence 2: MG05991.1	1077 aa
Sequence 3: FG07499.1	1154 aa
Sequence 4: AN1700.1	1144 aa
Alignment Length: 1223 aa
Number Identitical Residues: 532 aa
Alignment Score (without introns) 26410


MG05991.1 	-----------------------------------------------~------------
NCU09450.1	MPGLVTATGVLAFLADEEPELKVFALKTLNDDIDTVWTEVAGALTQI2------------
FG07499.1 	MPGIVSATGVLAFLTDEEPELKVFALQTLNDDIDTVWTEVAAVLTQI2------------
AN1700.1  	MVGLASAAGLVGFLSEPDSELKVFALQTLDSQIDLLWTEVVDAIPQI2RRLCGNGYQTNI
          	  .  :::.  .  :. ... .  : .: ... .   :.     ..     .... .:. 

MG05991.1 	------------------------------------------------------------
NCU09450.1	----EALYEDETFPERQLAALVLAKVYFHLQAYNESMTFALAAGPLFKLDAPSEFEETII
FG07499.1 	----EALYEDESFPERQLAALVLAKVYYHLQAYNDSMVFALAAGDLFKLDSPGEFEETII
AN1700.1  	QFLREALYEDEDFPERELAALVAAKVYYHLQEYHESMVFALGAGKLFNLDNGGEFEETII
          	.   .:  ...  .. . ::   :.     .   .:   : .:.   . .  .. ..:  

MG05991.1 	-~------MHHSPAK----PSKSDTSLPTLETTFAAGVTDPSALMSPTTPFSQSTLPSKS
NCU09450.1	S~KCIDQYIAVSSLH----HTPSKKDLPALETDLGSGAIDSSALISPTTPFSQSSVPSKS
FG07499.1 	S~KCVDQYIAVTAAKKAAPPASKNDDLPELATTFASGAEG--AVMSPTTPFSRTTLPPKS
AN1700.1  	A1KCVDTFISLSTSERPSAGDQPTNLNTAFPTSVEGASSTSASLTSPITPFSQSALPSKS
          	: .. .  :  :. . .:.        . : * . ..   .::: ** ****::::*.**

MG05991.1 	LLSRDST-DNTILDPTFQPATKKGRSGSIVQLPDQQTQKALQRVIERLFENCLKAGRYRQ
NCU09450.1	LLARASSDDNTILDPTFQP-TKETRSASIAQVHDTATQVALQNVIERLFESCLKEGKYRQ
FG07499.1 	LLSRDSI-DNTMLEATFQPSFKQFRSGSIAELPEQATD-SLQRVVDRLFESCLEKGRYRQ
AN1700.1  	LLSRQEV---TGVDGAHPGAEETGSNVEVPLVLKRGVQGQLKAVIERLFEQCFLQKRYRQ
          	**:* .    * :: :.  : :   . .:  : .  .:  *: *::****.*:   :***

MG05991.1 	VVGIAVEAKNLDVLRGVIKRASDDEKRTKGKSQESIQSPGDELMEYALGICMDIVQERSL
NCU09450.1	VVGIAVEAKNLDVLRRVIKRANQDVKSGKAKVQEGSQGPAEELMDYALSICMDIVQERGF
FG07499.1 	VVGIAVEAKNLAVLRSVIKRASDDEKKAKSNPLESSPGPAEDLIEYTLGICMDIVQERGF
AN1700.1  	VIGIAIEAKDLEVLRTAIMRASEDAKQQNGESRQS-----EELMEYVLDICMGVVQERGF
          	*:***:***:* *** .* **.:* *  :.:  :.     ::*::*.*.***.:****.:

MG05991.1 	RTE~ILRLILSLLNDIPNPDYFAIARCVVYLNADEQASTMLRKLVDNGDVNSIASAYQIA
NCU09450.1	RTE~ILRLILDLLNDIPNPDYFAIAKCVVYLDSDEEASRMLRQLVAKGEQDSIAIAYQIA
FG07499.1 	RTE~ILRLILDLLNEIPNPDYFAIAKCVVYLNSDEEASRMLRNLVEKADRSSIAIAYQIA
AN1700.1  	RNE0ILKLILELLNEIPSPDYFAIAKCVVYLNEHSMASVILRQLVEKGDARSLAVAYQIS
          	*.* **:***.***:**.*******:*****: .. ** :**:** :.:  *:* ****:

MG05991.1 	FDLYDNGTQEFLGKVIKSLPSGEPPKKPEAEKPEGAEPTEDAPLLEDAEESTPDDAEEEL
NCU09450.1	FDLYDNSTQEFLGKVIKALPSGEPAK-PKPAEGSNDAAAEREPLLEGQESS-----EEEL
FG07499.1 	FDLYDNGTQGFLGNVLASLPAGEPAK--SAVDENGVQPTESESLLQNQQDS----PENEL
AN1700.1  	FDLYDNSTQEFLQKVHQEISELVPEK-TEDSETKEEEPKESDALLDNQSSSIGANADSKV
          	******.** ** :*   :.   * * ..  . .   . *  .**:. ..*   ..:.::

MG05991.1 	PEEVAKVFRNIRTILDGSKTIKLNLEFLYRNNHTDLSILNKVRESLEGRNSIFHTAVTFC
NCU09450.1	PEEVAKVYKNIRTILDGSKVIKLNLEFLYRNNHADMSILNKVRDSLDGRNSIFHSAVTFC
FG07499.1 	SEPLRKAYSNIRAILDGSKTIRLNLEFLYRNNRTDLSILNKVRDSLEGRNSIFHTAVTFC
AN1700.1  	SAEARTAFKNILDILDGIKTIQLNMEFLHRNSRVDIAILNKLRDTLEPRNSIFHSAITIS
          	.    ..: **  **** *.*:**:***:**.:.*::****:*::*: ******:*:*:.

MG05991.1 	NAFMNAGTTHDKFFRDNLEWLGKAVNWSKFTATAALGVIHRGNLTQSRKLLEPYLPKSGG
NCU09450.1	NAFMNAGTTNDRFFRDNLEWLGRAVNWSKFTATAALGVLHRGNVTQSRKLLEPYLPRPSG
FG07499.1 	NAFMNQGTTNDKFFRDNLEWLGKAVNWSKFTATAALGVIHRGNLSQSRKLLEPYLPRQGG
AN1700.1  	NGFMYAGTAQDKFFRDNLEWLGKAVNWSKFTATAALGVIHRGNLSQGQRLLQPYLPKENS
          	*.**  **::*:**********:***************:****::*.::**:****: ..

MG05991.1 	LS----SGSTFSQGGALYAYGLIHANHGADALDYLRSMFTGHDDEVIRHGGALGLGIAGM
NCU09450.1	VS----TGSVYSQAGALYAYGLIHANHGADALDYLHQQFKDADQEVIQHGGALGLGIAGM
FG07499.1 	LS----SGSIFSQGGALYAYGLIHANHGAEALDYLKEQFGQAEEEVVQHGGALGLGIAGM
AN1700.1  	IDGVGNSGSLYSQGGSLYALGLIYANHGGMAVDYIRDQFKKATEEVIQHGGALGLGVAGM
          	:.. ..:** :**.*:*** ***:****. *:**::. *    :**::********:***

MG05991.1 	ATGDEEMFEELKNVLWQDSALNGEAVGLAMGLVMLGTGNANALKTMFQYAHDTTHEKIVR
NCU09450.1	GTGDQKIIEDLKEALYSDSALNGEAVGAAMGLIMLGTGHAATIDAMFTYAHETQHEKIVR
FG07499.1 	ATGDMDIYDKLREVLFQDSALNGEAVGLAMGLVMLGTGHARALEDMITYAHETTHEKILR
AN1700.1  	ATGDEGIYDDLRNVLYTDSALNGEAVGLAMGLVMLGTGNMKVLEDMIQYAHDTQHEKIVR
          	.***  : :.*::.*: ********** ****:*****:  .:. *: ***:* ****:*

MG05991.1 	GCALGMALIMFGRQEGADVIIEALLADPDPTLRYGGILTIAMAYCGTGSNKAIRKLLHTA
NCU09450.1	GIALGIALIMFGQQEGADRTIQLLLANPDPILRYGGVLTLAMAYCGTGSNKAIRKLLHLA
FG07499.1 	GLSIGMALIMFGRQEGADVLIEGLLNDPDPTLRYGGIMTVALAYCGTGSNKAIRKLLHTA
AN1700.1  	GLAMGMALIMYGRQEAADELINGLLGDPDPTLRYGGIMTIALAYCGTSSNKAVRKLLHVA
          	* ::*:****:*:**.**  *: ** :*** *****::*:*:*****.****:***** *

MG05991.1 	VSDVNDDVRRIAVMSLGFILFRKPGSVPRMVELLSESYNPHVRYGSAMALGISCAGTGLD
NCU09450.1	VSDVNDDVRRVAVMSLGFILFRKPGSVPRMVELLAESYNPHVRYGSAMALGIACAGTGLD
FG07499.1 	VSDVNDDVRRIAVMSLGFILFRKPGSVPRMVELLSESYNPHVRYGSAMALGISCAGTGLD
AN1700.1  	VSDVNDDVRRVAVLSLGFILFRKHQSVPRMVELLSESYNPHVRYGAAMALGISCAGTGLD
          	**********:**:*********  *********:**********:******:*******

MG05991.1 	EAIDLLEPMMKDPTDFVRQGALISLAMIMVQQNEAMNPKVASIRKTLKKVVGDRHEDAMT
NCU09450.1	EAIDLLEPMMKDPTDFVRQGALIALSMILVQQNEVMNPKVAAIRKTLKKVVSDRHEEQMT
FG07499.1 	EAIDLLEPMMKDPTDFVRQGALISLAMILVQQNEIMNPKVASIRKTLKKVVGDRHEDAMT
AN1700.1  	EAIDLLEPMLKDSTDFVRQGALIALAMVLVQQNEAMNPRVSSLRKTMMKMVSDRHEDAMA
          	*********:**.**********:*:*::***** ***:*:::***: *:*.****: *:

MG05991.1 	KFGASLALGIIDAGGRNCTIGLQTQTGNLNMPGIVGMAIFTQYWYWFPFTHFLSLSFSPT
NCU09450.1	KFGAALALGIIDAGGRNCTIGLQTQTGNLNMAGIVGMAVFTQYWYWFPFTHFLSLSFVPT
FG07499.1 	KFGAALALGIIDAGGRNCTIGLQTQTGNLNMAGIVGMAVFTQYWYWFPFTHFLSLSFSPT
AN1700.1  	KFGCAIALGIIDAGGRNCTISLQTQTGNLNMPGIVGAAVFLQYWYWFPLTHFLSLSFAPT
          	***.::**************.**********.**** *:* *******:******** **

MG05991.1 	SIIGLDHDLEIPNFKFHCAARPSLFDYPPEQEVKTEEGPALIATAVLSTTAQAKRRAQKK
NCU09450.1	SIIGLDSDLAIPNVKFHCATRPSLFDYPPEQEVKVEEGPALVTTAVLSTTAQAKRRAQKK
FG07499.1 	SIIGLDHDLEIPDTKFHCATRPSLFDYPPEQEVKTEEGPALIATAILSTTAQAKRRAQKK
AN1700.1  	SVIGVDQKLEVPFFKFHSNTRPSLFDYPPEQQVKAEEAPEKVKTAVLSTTALAKRRALRR
          	*:**:* .* :*  ***. :***********:**.**.*  : **:***** ***** ::

MG05991.1 	ASAQRRESMDIDSPAVSKPVVAEAG-DKMDVDDDKK--AKSDDSKDKKDGESAVEKEGSA
NCU09450.1	EKAQRRESMDIDTPAVSKTASQPAAEDKMDIDDDKKAAAKPEESKEKKEGESG-EKEATG
FG07499.1 	ERAQRRESMDIDS------APAKPSGDKMDVDEDKK----TDETNEKKE---EQEKEAAT
AN1700.1  	EKQHRRESMDVDQTP-TTPKVADQMPEKMETDEDKG-------DEELKEG----DKEAVE
          	   :******:* .. :..       :**: *:**         :: *:.    :**.  

MG05991.1 	APETKKKAEREKVGYEIENISRVLPGQLKFISFPAGRYKPVKK~PTGGPLLLQDTQPDEP
NCU09450.1	ATDAKKKAEKEKVGYEIENMSRILPGQLKYISFPAGRYKPVKK0PTGGPLLLFDTQPSEA
FG07499.1 	STDTKKKPEKEKIGYDIENMTRVLPGQLKYISFNSGRYKPVKK0PTGGPLLLIDGEPEEE
AN1700.1  	G--QKKKAEREKVGYELENMSRVLPAQLKYLTFPDPRYEPVKR0PTGGVVVVLDKTPEEP
          	.   ***.*:**:**::**::*:**.***:::*   **:***: **** ::: *  *.* 

MG05991.1 	KSLIEEKLKKVTVEKAPVAGAQAGSSARDGGRMGG-----LMTDQQALIRAAQQIASGVG
NCU09450.1	KSLVEEKLNKVTTERAPVAGAGASGASDGTPRLSGGAAQSLLSQLQGTGAFNRSSMRELS
FG07499.1 	KALLEEKLKKVTTERAPVAGQQNARGGRSGGPGRS-----LLDGITDAQRGGPS-SAMLG
AN1700.1  	RDVIELKASKETKQPALATESAVPADLQAR-----------LAEFTDPSR----------
          	: ::* * .* * : * .:                      :                  

MG05991.1 	AGGDDNWREALGRGGRAGESELFRQLLGGRSQSGAAGVSGGDQRTGAAAAAGVLTAVDED
NCU09450.1	ELLQLGQRMEQDASAGLGES-RARGAGGAAAGSGTGASATAGSGAGAAAAAAVLNAVDED
FG07499.1 	QLLRNSGRSPFG-------------------LLDADPQTPGGNASGAAAAAGVLTAVDED
AN1700.1  	-LLATPRR-----------------------------PENPLSGSGAAAAAGVLTAVDED
          	       *                                  . :******.**.*****

MG05991.1 	GEDDEEASVPRDFEYFTDNEDEEL---
NCU09450.1	AEGDEEAPTPAEFEYFTDAEADDEE--
FG07499.1 	S-DDEEAKTPNEFEYFTDAEDDDDDDE
AN1700.1  	E-DGEEAPVPEEFGYTSEGEAEE----
          	  ..*** .* :* * :: * ::
```
